# Time-Dependent Anabolic Response of hMSC-Derived Cartilage Grafts to Hydrostatic Pressure

**DOI:** 10.1155/2023/9976121

**Published:** 2023-08-23

**Authors:** Farhad Chariyev-Prinz, Nuno Neto, Michael G. Monaghan, Daniel J. Kelly

**Affiliations:** ^1^Trinity Centre for Biomedical Engineering, Trinity Biomedical Sciences Institute, Trinity College Dublin, Dublin, Ireland; ^2^Department of Mechanical, Manufacturing and Biomedical Engineering, School of Engineering, Trinity College Dublin, Dublin, Ireland; ^3^Advanced Materials and Bioengineering Research Centre (AMBER), Royal College of Surgeons in Ireland and Trinity College Dublin, Dublin, Ireland

## Abstract

It is generally accepted that the application of hydrostatic pressure (HP) is beneficial for MSC chondrogenesis. There is, however, evidence to suggest that the timing of application might determine its impact on cell fate and tissue development. Furthermore, understanding how the maturity of engineered cartilage affects its response to the application of HP can provide critical insight into determining when such a graft is ready for *in vivo* implantation into a mechanically loaded environment. In this study, we systematically examined chondrogenic maturation of hMSCs over 35 days in the presence of TGF-*β*3 *in vitro*. At specific timepoints, the response of hMSCs to the application of HP following the removal of TGF-*β*3 was assessed; this partially models conditions such grafts will experience *in vivo* upon implantation. In free swelling culture, the expression of chondrogenic (*COL2A1* and *ACAN*) and hypertrophic (*COL10A1*) markers increased with time. At early timepoints, the expression of such markers continued to increase following TGF-*β*3 withdrawal; however, this was not observed after prolonged periods of chondrogenic priming (35 days). Interestingly, the application of HP was only beneficial after 35 days of chondrogenic priming, where it enhanced sGAG synthesis and improved key chondrogenic gene ratios. It was also found that HP can facilitate a metabolic shift towards oxidative phosphorylation, which can be viewed as a hallmark of successfully differentiating MSCs. These results point to the importance of mechanical loading as a key stimulus for maintaining a chondrogenic phenotype once MSCs are removed from chemically defined culture conditions.

## 1. Introduction

Articular cartilage (AC) is a highly specified tissue that covers the ends of bones within the diarthrodial joints. Its unique biochemical composition is responsible for its load bearing and low friction properties during articulation. Although evolutionary designed to operate within mechanically demanding environments, AC possesses very limited intrinsic regenerative capacity, which can be linked to its avascular, alymphatic, and aneural nature[1]. Additionally, damage and injuries to AC are strongly associated with the development of posttraumatic osteoarthritis (OA) or secondary OA. This progressive degenerative disease represents a significant societal and financial burden worldwide, and the development of an effective therapy represents a great challenge to this day [2].

AC tissue engineering (TE) represents a promising strategy to circumvent some of the shortcomings of conventional therapies for joint regeneration such as autologous chondrocyte implantation (ACI), microfracture, or mosaicplasty [3]. Drawbacks associated with ACI such as donor site morbidity and cell availability could be negated by the use of more readily available undifferentiated mesenchymal stem/stromal cells (MSCs), which in combination with appropriate scaffolds and growth factors such as TGF-*β*3 can be steered to differentiate into chondrocyte-like cells and deposit cartilage-specific extracellular matrix (ECM) components [4]. In this context, although the role of growth factors such as TGF-*β*3 during chondrogenic induction of MSCs *in vitro* is well established, the effect of removing supraphysiological levels of such factors (e.g., upon implantation *in vivo*) on engineered graft development remains poorly understood. Therefore, it is paramount to identify conditions that facilitate the development of a stable chondrocyte-like phenotype in chondrogenically primed MSCs. In addition to phenotype maintenance, chondrogenically primed MSCs must respond anabolically to joint-like mechanical loading. However, it remains unclear when TGF-*β*3-stimulated MSCs adopt such a mechanoresponsive phenotype.

Mechanical stimulation is crucial to skeletogenesis and normal joint development [5]. Interestingly, fracture healing, which is a highly complex regenerative process, is known to recapitulate aspects of embryonic development, where a combination of mechanical forces, specialized cells, and secreted factors drive regeneration of the tissue, albeit the interplay of individual factors is yet to be fully elucidated [6]. Nevertheless, the correct timing of load application has been shown to play a key role during regeneration in various fracture healing models, where delayed application of mechanical load has been shown to be more beneficial in comparison with immediate mechanical stimulation for fracture healing [7, 8]. Similarly, for AC regeneration, the *in vivo* environment appears to orchestrate how progenitor cells differentiate. For example, MSCs implanted into cartilage defects in mini-pigs have been shown to be less hypertrophic than control cells maintained *in vitro* [9], pointing to the importance of joint-specific cues in maintaining a cartilage phenotype. In another study, implantation of predifferentiated human MSCs in a pig model revealed that longer predifferentiation periods *in vitro* resulted in better collagen type II and sGAG deposition *in vivo* [10]. Cumulatively, these studies indicate that joint-specific biomechanical stimuli are integral to directing the differentiation of stem/progenitor cells and the engineering of functional cartilage grafts; however, the maturity such engineered cartilage tissues needs to achieve to respond anabolically to biophysial cues remains poorly understood.

Hydrostatic pressure (HP) bioreactors have been employed in the studies to emulate the mechanical environment within the joints and to examine how such cues affect the differentiation of MSCs during chondrogenesis *in vitro* [11]. It is well established that the appropriate application of HP can benefit chondrogenic differentiation of MSCs *in vitro* and facilitates cartilage-specific ECM deposition, albeit the extent of benefits varies greatly between the experiments [12, 13]. Generally, HP has been shown to benefit chondrogenesis when applied during or after various chondrogenic priming periods in the presence of TGF-*β*3 [11]. It is also possible to use such bioreactors to subject engineered cartilage tissues to joint-like loading conditions, in combination with the removal of TGF-*β*3 from the culture media, as a model system to better understand how these engineered cartilage grafts will respond to *in vivo*-like mechanical loads once they are removed from chondrogenic culture conditions [14, 15]. We have previously shown that chondrogenically primed human MSCs can respond positively to the application of HP [16]. What remains unclear is how long such engineered grafts need to be maintained in the presence of chondrogenic growth factors before they can mount an anabolic response to the application of HP in the absence of such biochemical cues.

The goal of this study was to use a HP bioreactor as an *in vitro* platform to model and understand how chondrogenically primed hMSCs would respond to joint-like mechanical loading *in vivo*. Specifically, we sought to explore how the phenotype of hMSCs, which were first chondrogenically primed for different periods of time in the presence of TGF-*β*3, would change in response to the application of HP. By investigating the anabolic response of hMSCs at different timepoints of chondrogenic differentiation, we aimed to identify the degree of “maturation” necessary for cartilage grafts to potentially respond favourably to the *in vivo* joint environment. It this way, we hope to demonstrate how such *in vitro* bioreactor platforms can be utilized for screening of cartilage grafts prior to implantation, which might reduce the number of AC TE grafts that fail *in vivo* [17].

**Figure 1:**
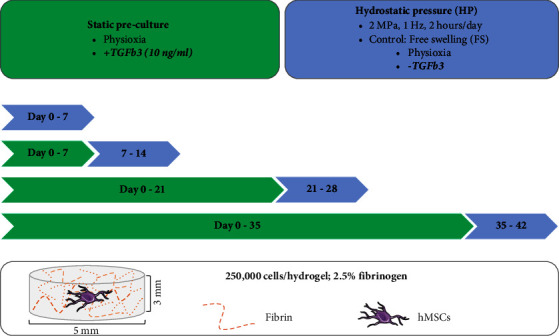
Experimental design. Fibrin-encapsulated hMSCs were cultured in chondrogenic media (10 ng/ml TGF-*β*3) for specified periods, and subsequently, HP was applied for a week in the absence of TGF-*β*3.

To this end, hMSCs were cultured for either 7, 21, or 35 days in the presence of 10 ng/ml TGF-*β*3, at which point this growth factor was removed from the media and the constructs were stimulated with 2 MPa of HP for an additional 7 days. Using this platform, we sought to determine how long hMSCs need to be primed *in vitro* before they respond anabolically to joint-like loading (Figure 1). To better mimic the *in vivo* scenario, where chondrocytes are exposed to oxygen levels between 1-5% *in vivo*, 5% O_2_ tension was employed during cell expansion and differentiation, conditions which have previously been shown to support more robust chondrogenesis of MSCs [18–21].

## 2. Materials and Methods

### 2.1. Cell Isolation and Expansion

Human bone marrow-derived MSCs (hBMSCs) were isolated from bone marrow aspirates (Lonza) and expanded in high-glucose Dulbecco's modified Eagle's medium (DMEM) GlutaMAX supplemented with 10% v/v FBS, 100 U/ml penicillin, 100 mg/ml streptomycin (Gibco, Biosciences), and 5 ng/ml human fibroblast growth factor-2 (FGF-2; PeproTech) at 37°C, 95% humidity, 5% CO_2_, and 5% O_2_. Expansion of cells was initiated at a density of 5000 cells/cm^2^ in T175 flasks; cells were trypsinized once 80% confluency was reached. All cells were used at passage 4 if not mentioned otherwise. MSCs from one donor have been used consistently throughout the study.

### 2.2. Preparation and Culture of Cellular Fibrin Hydrogels

Fibrinogen (Sigma) was dissolved in a NaCl (19 mg/ml; Sigma)-aprotinin (Nordic Pharma) solution and combined with cells resuspended in a thrombin solution (5 U/ml; Sigma); the solution was then polymerised in silicon moulds (60 *µ*l/well; *Ø* = 5 mm; *h* = 3 mm) for 40 minutes at 37°C. Cellular hydrogels with 250,000 cells/gel were cultured in 24-well plates with chondrogenic media (CDM+) containing DMEM supplemented with 100 U/ml penicillin, 100 mg/ml streptomycin (both Gibco), 100 mg/ml sodium pyruvate, 40 mg/ml L-proline, 50 mg/ml L-ascorbic acid-2-phosphate, 4.7 mg/ml linoleic acid, 1.5 mg/ml BSA, 1 X insulin-transferrin-selenium, 100 nM dexamethasone (all Sigma-Aldrich), aprotinin (100 KIU/ml; Nordic Pharma), and 10 ng/ml TGF-*β*3 (PeproTech) [14]. Each hydrogel was cultured in a 24‐well plate in 2 ml media, which was exchanged twice a week until application of HP.

### 2.3. Hydrostatic Pressure Application

After preculture period in well plates, hydrogels were transferred into gas permeable and watertight cell culture bags (OriGen Biomedical). Heat-sealed bags were then filled either with CDM + without TGF-*β*3. If not mentioned otherwise, each bag containing 4 ml media/hydrogel was not exchanged during the week of loading to reduce the risk of contamination. For hydrostatic pressure (HP) application, cell culture bags were transferred into a custom-build bioreactor within a 37°C incubator as described previously [15]. Cells were subjected to HP at 2 MPa at 1 Hz for 2 hours daily. The free swelling controls were placed in the same incubator for the duration of stimulation. After stimulation, all bags were returned to culture incubators (37°C, 5% CO_2_, and 5% O_2_).

### 2.4. Gene Expression Analysis

Total RNA extraction was performed using TRIzol™ reagent (Thermo Fisher) in accordance with the manufacturer's instruction. Prior to isolation, samples were physically disrupted with pestles to facilitate equal disruption and dissolution of cells and tissues. After elution and RNA quantification (NanoDrop), equal amounts of RNA for each sample were reverse-transcribed to cDNA using High Capacity cDNA Reverse Transcription Kit (Applied Biosystems™). Reverse-transcribed product was quantified with Qubit™ ssDNA Assay kit (Thermo Fisher), and 10 ng cDNA of each sample was used for real-time PCR. Amplification was performed using SYBR™ Select Master Mix and 7500 Fast System (Applied Biosystems™). B2M functioned as a housekeeping gene and amplification of target genes was quantified using the 2^−ΔΔCt^ method. KiCqStart® SYBR® Green predesigned primers were purchased from Sigma (see Table 1).

### 2.5. Quantitative Biochemical Analysis

Halved samples were digested overnight at 60°C in digesting buffer containing 50 mM Na_2_HPO_4_, 50 mM NaH_2_PO_4_, 5 mM EDTA, 10 mM L-cysteine, and 3.88 U/ml papain at pH 6.4 (all Sigma-Aldrich). DNA content was assessed with Hoechst 33258 based quantification kit as per the manufacturer's protocol (Sigma-Aldrich). Sulfated glucosaminoglycan content (sGAG) was quantified with 1,9-dimethylene blue (DMMB) at pH 1.5; metachromatic changes of DMMB in the presence of sGAG were monitored at 530 and 590 nm. Shark chondroitin sulfate was used as standard (Sigma-Aldrich). For collagen quantification, the digest was further hydrolyzed with 38% HCl overnight; samples were then oxidized with chloramine-T (41 mM) to obtain pyrrole-2-carboxylate. Indoles were quantified with Ehrlich's reagent containing 4-dimethylbenzaldehyde (2 M) at 570 nm. Trans-4-hydroxy-L-proline (Sigma-Aldrich) was used as a standard, and collagen content was calculated using hydroproline: collagen ratio of 1 : 7.69 [22]. All spectrophotometric analysis was performed using a Synergy HT multidetection plate reader (BioTek Instruments, Inc.).

### 2.6. Histological Analysis

Halved samples were fixed in 4% paraformaldehyde overnight at 4°C. Upon fixation, samples were dehydrated in a series of ethanol solutions (50–100%). Subsequently, samples were cleared with xylene and infiltrated with paraffin wax. All steps from dehydration to infiltration were performed in an automated fashion (Leica Biosystems). Samples were then embedded in paraffin blocks for microtome slicing (5 *µ*m, Leica Biosystems). All histological stains were performed with an autostainer (Leica). For sGAG determination, alcian blue stain (AB) at pH 1.0 (1% w/v alcian blue 8GX in 0.1 M hydrochloric acid (HCl)) was used; nuclear fast red (0.1% w/v) was used as counterstain. For collagen deposition, picrosirius red (PR) 0.1% w/v was used. All stained slides were imaged with Aperio ScanScope (Leica Biosystems).

### 2.7. Immunohistochemistry

Hyaluronidase (Sigma-Aldrich) 1% w/v and pronase (Merck) 3.5 U/ml were used for antigen retrieval (25 minutes at 37°C, respectively). Sections were blocked in the presence of 10% v/v goat serum and 1% w/v BSA (Sigma-Aldrich) at RT and incubated with mouse primary antibodies overnight at 4°C. Sections were blocked in the presence of 10% v/v goat serum and 1% w/v BSA (Sigma-Aldrich) at RT and incubated with mouse primary antibodies against Col10a1 (AB49945, 1 : 200) overnight at 4°C. Goat anti-mouse IgM (AB150121) was used as secondary antibody for Col10a1 sections (4 hours at RT). Goat anti-mouse IgM (AB150121) was used for Col10a1 sections (4 hours at RT); nuclei were counterstained with DAPI (2 *μ*g/mL, Sigma-Aldrich). All samples were mounted with ProLong™ Gold Antifade (Invitrogen). Presence of collagen type *X* was visualized using Leica SP8 scanning confocal microscope. Representative images are shown.

Two-photon fluorescence lifetime imaging microscopy (2-P FLIM) was performed on a custom multiphoton system as previously described [23, 24]. Briefly, two-photon excitation of nicotinamide adenine dinucleotide phosphate (NAD(P)H) fluorescence was performed with 760 nm excitation wavelength. A 455/90 nm bandpass filter was used to isolate NAD(P)H fluorescence signal. 512 × 512 pixel images were acquired with a pixel dwell time of 3.81 *μ*s and 30-second collection time. At least 3 images for each sample were acquired.

### 2.8. Statistical Analysis

Statistical analysis was performed using GraphPad (GraphPad Software, USA). All experiments were performed with at least three replicate samples per condition. The significance of mean differences among two conditions was evaluated using unpaired two-tailed Student's *t*-test. The significance of differences among groups was performed using one-way analysis of variance (ANOVA) with a post hoc Tuckey's multiple comparison test. Two-way analysis of variance (ANOVA) with a post hoc Tuckey's multiple comparison test was used to analyse the statistical differences among groups with two independent variables (e.g., stimulation and duration). Numerical values were presented as mean ± standard deviation. Values of *p* ≤ 0.05 were considered significant.

## 3. Results

### 3.1. hMSC Differentiation and Tissue Maturation over 35 Days in Chondrogenic Media

**Figure 2:**
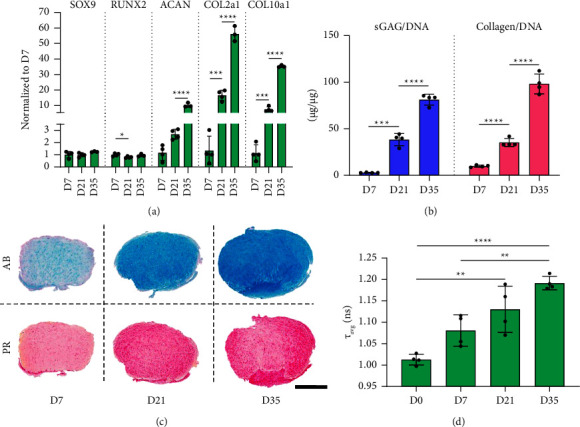
Chondrogenic differentiation of hMSCs over 35 days. (a) Gene expression analysis of chondrogenic (*SOX9*, *ACAN*, and *COL2A1*) and hypertrophic (*RUNX2* and *COL10A1*) markers. (b) Biochemical sGAG and collagen content analysis. sGAG content was determined with DMMB-based assay; total collagen was determined via hydroxyproline-based quantification assay. All values were normalized to total DNA. (c) Histological assessment of hydrogels with alcian blue (AB) and picrosirius red (PR); scale bar = 1 mm. (d) *τ*_avg_ values over the course of 35 days during chondrogenic differentiation. Significant differences among groups are reported using one-way analysis of variance (ANOVA) with a post hoc Tukey's multiple comparison test. All data were represented as mean ± SD; technical replicates *n* ≥ 4, ^*∗*^*p* ≤ 0.05, ^*∗∗*^*p* ≤ 0.01, ^*∗∗∗*^*p* ≤ 0.001, and ^*∗∗∗∗*^*p* ≤ 0.0001.

The chondrogenic differentiation of hMSCs (encapsulated within 2.5% fibrin hydrogels) in response to TGF-*β*3 stimulation over 35 days in static culture conditions was first assessed. A continued increase in the expression of the chondrogenic markers *ACAN* and *COL2A1*, as well as of the hypertrophic marker *COL10A1*, was observed over the course of 35 days (Figure 2(a)). No significant changes in the expression of the transcriptional factors *SOX9* and *RUNX2* could be observed after 7 days of culture. A time-dependent increase in matrix accumulation was also observed, with sulfated glucosaminoglycans (sGAGs)/DNA levels reaching 81.8 (*µ*g/*µ*g) and collagens/DNA 94.6 (*µ*g/*µ*g) values at day 35 (Figure 2(b)). Histological analysis further confirmed robust chondrogenic differentiation, with the most intense staining for alcian blue and picrosirius red observed at day 35 (Figure 2(c)). Lastly, protein-bound NAD(P)H was quantified using Fluorescence Lifetime Imaging Microscopy (FLIM) to assess metabolic changes as function of cell maturation (Figure 2(d)). Over the course of 35 days, *τ*_avg_ values steadily increased, thus indicating overall increases of protein-bound NAD(P)H, with significant increases at day 35 compared to day 0 and 7. This points to cells adopting OxPhos (oxidative phosphorylation) metabolic profile with time in culture.

### 3.2. The Effect of Withdrawing TGF-*β*3 on the Phenotype of Chondrogenically Primed hMSCs

**Figure 3:**
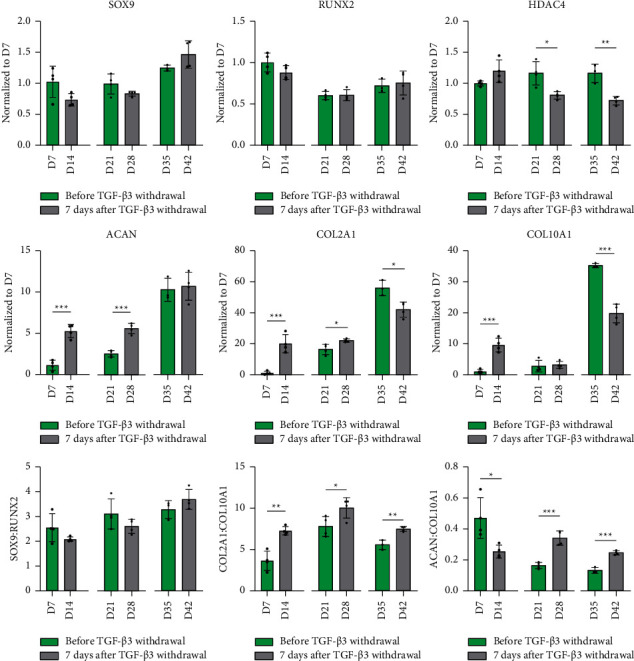
Gene expression analysis. Influence of TGF-*β*3 priming and subsequent removal on the expression of chondrogenic and hypertrophic markers. Relative gene expression of chondrogenic (*SOX9, COL2A1, ACAN,* and *HDAC4*) and hypertrophic (*RUNX2* and *COL10A1*) markers has been normalized to expression levels at day 7 and determined using the 2^−ΔΔCt^ method. Gene expression ratios for *SOX9/RUNX2*, *COL2A1/COL10A1*, and *ACAN/COL10A1* were determined using 2^−ΔCt^ values. Housekeeping gene: B2M. Significant differences are reported using unpaired two-tailed Student's *t*-test, which was employed to reveal the effect of TGF-*β*3 at one specific timeframe. All data were represented as mean ± SD; technical replicates *n* ≥ 3, ^*∗*^*p* ≤ 0.05, ^*∗∗*^*p* ≤ 0.01, and ^*∗∗∗*^*p* ≤ 0.001.

Having established the time-course of chondrogenic differentiation in this fibrin hydrogel system, we next examined how the removal of TGF-*β*3 from the culture media affects the expression of key chondrogenic and hypertrophic markers (Figure 3). The removal of TGF-*β*3 after 35 days of priming resulted in a downregulation of *COL2A1* and *COL10A1* by day 42; removal of the growth factor at earlier timepoints had no negative effect on the expression of these genes. The expression of *HDAC4* (histone deacetylase 4 is associated with the suppression of hypertrophic markers [25]) reduced when TGF-*β*3 was removed after 21 and 35 days of priming. For all examined timepoints, the removal of TGF-*β*3 did not result in a reduction in the expression of *ACAN* over the following 7 days of culture; in fact, the expression of *ACAN* continued to increase after the removal of this growth factor at either day 7 or 21. The ratio of *COL2A1/COL10A1* expression also increased when TGF-*β*3 was removed from the media after 7 or 35 days of priming.

**Figure 4:**
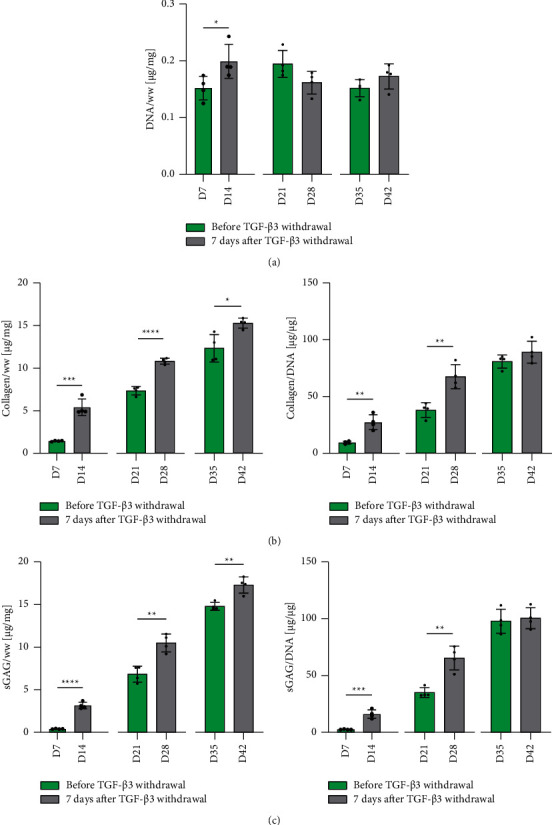
Biochemical content analysis. Influence of TGF-*β*3 priming and subsequent removal on deposition of ECM components. (a) DNA content normalized to wet weight (ww) was determined using Hoechst bisbenzimide H 33258 quantitation assay. (b) Collagen content analysis. Total collagen was determined via hydroxyproline-based quantification assay and normalized to wet weight as well as total DNA. (c) sGAG content analysis. sGAG content was determined with DMMB-based assay and normalized to wet weight as well as DNA content. Significant differences are reported using unpaired two-tailed Student's *t*-test, which was employed to reveal the effect of TGF-*β*3 at one specific timeframe. All data were represented as mean ± SD, technical replicates *n* ≥ 3, ^*∗*^*p* ≤ 0.05, ^*∗∗*^*p* ≤ 0.01, ^*∗∗∗*^*p* ≤ 0.001, and ^*∗∗∗∗*^*p* ≤ 0.0001.

Biochemical assays were used to evaluate how the removal of TGF-*β*3 influences overall levels of ECM deposition. No major changes in the overall DNA content over the course of the experiment could be observed **(**Figure 4(a)). Removal of TGF-*β*3 did not negatively affect collagen/DNA or sGAG/DNA deposition compared to the timepoint when it was removed (Figures 4(b) and 4(c)**)**. Collagen deposition continued to increase following the removal of TGF-*β*3 from the media on per cell basis at day 7 or day 21, with similar trends observed for sGAG deposition.

### 3.3. HP Does Not Initiate Robust Chondrogenesis of hMSCs in the Absence of Exogenous Growth Factors

**Figure 5:**
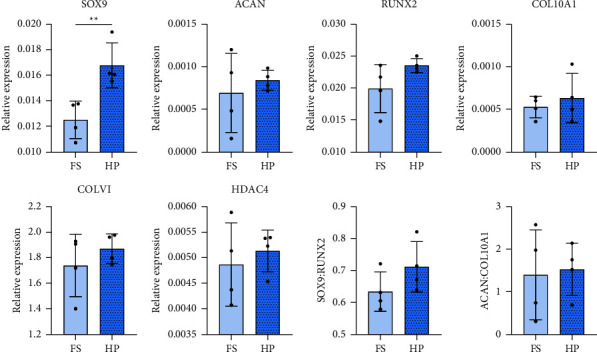
Gene expression analysis of hMSCs subjected to HP in absence of TGF-*β*3 after 7 days. Relative expression levels of chondrogenic (SOX9 and ACAN), hypertrophic (RUNX2 and COL10A1) marker and COLVI and HDAC4 genes were determined using the 2^−ΔCt^ method. Gene expression ratios for SOX9/RUNX2 and ACAN/COL10A1 were determined using 2^−ΔCt^ values. Housekeeping gene: B2M. Significant differences are reported using unpaired two-tailed Student's *t*-test. All data were represented as mean ± SD; technical replicates *n* = 4, and ^*∗∗*^*p* ≤ 0.01.

Next, the ability of hydrostatic pressure to induce chondrogenic differentiation in absence of TGF-*β*3 was investigated. Upon encapsulation of hMSCs in 2.5% fibrin hydrogels, they were directly mechanically stimulated without any other soluble cues to direct differentiation. Gene expression analysis of chondrogenic and hypertrophic markers revealed a significant upregulation in the expression of the *SOX9* transcription factor due to the application of HP **(**Figure 5**)**. Despite this, none of the other examined chondrogenic genes were significantly affected by the application of HP.

### 3.4. The Influence of HP on Chondrogenesis of hMSCs following Different TGF-*β*3 Priming Periods

**Figure 6:**
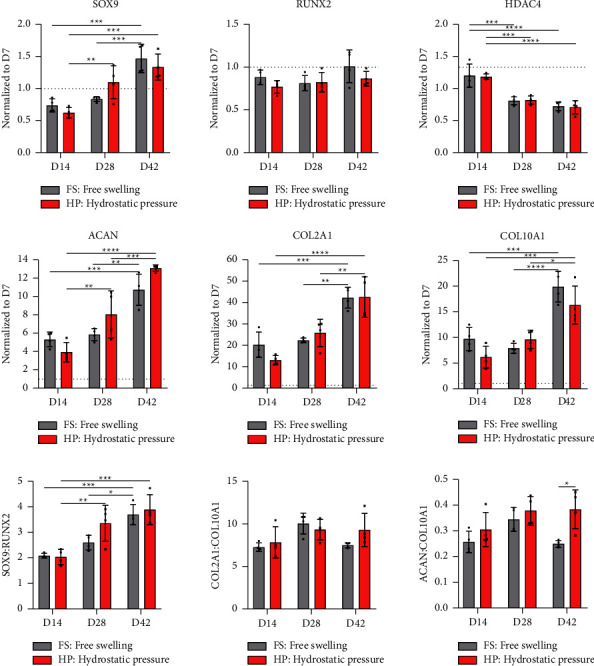
Gene expression analysis. Influence of the priming period on mechanoresponsiveness of hMSCs. Relative gene expression of chondrogenic (*SOX9, COL2A1, ACAN,* and *HDAC4*) and hypertrophic (*RUNX2* and *COL10A1*) markers has been normalized to expression levels at day 7. Relative expression levels were determined using the 2^−ΔΔCt^ method. Gene expression ratios for *SOX9/RUNX2*, *COL2A1/COL10A1*, and *ACAN/COL10A1* were determined using 2^−ΔCt^ values. Housekeeping gene: B2M. Significant differences are reported using two-way analysis of variance (ANOVA) with a post hoc Tukey's multiple comparison test. All data were represented as mean ± SD; technical replicates *n* ≥ 3, ^*∗*^*p* ≤ 0.05, ^*∗∗*^*p* ≤ 0.01, ^*∗∗∗*^*p* ≤ 0.001, and ^*∗∗∗∗*^*p* ≤ 0.0001.

**Figure 7:**
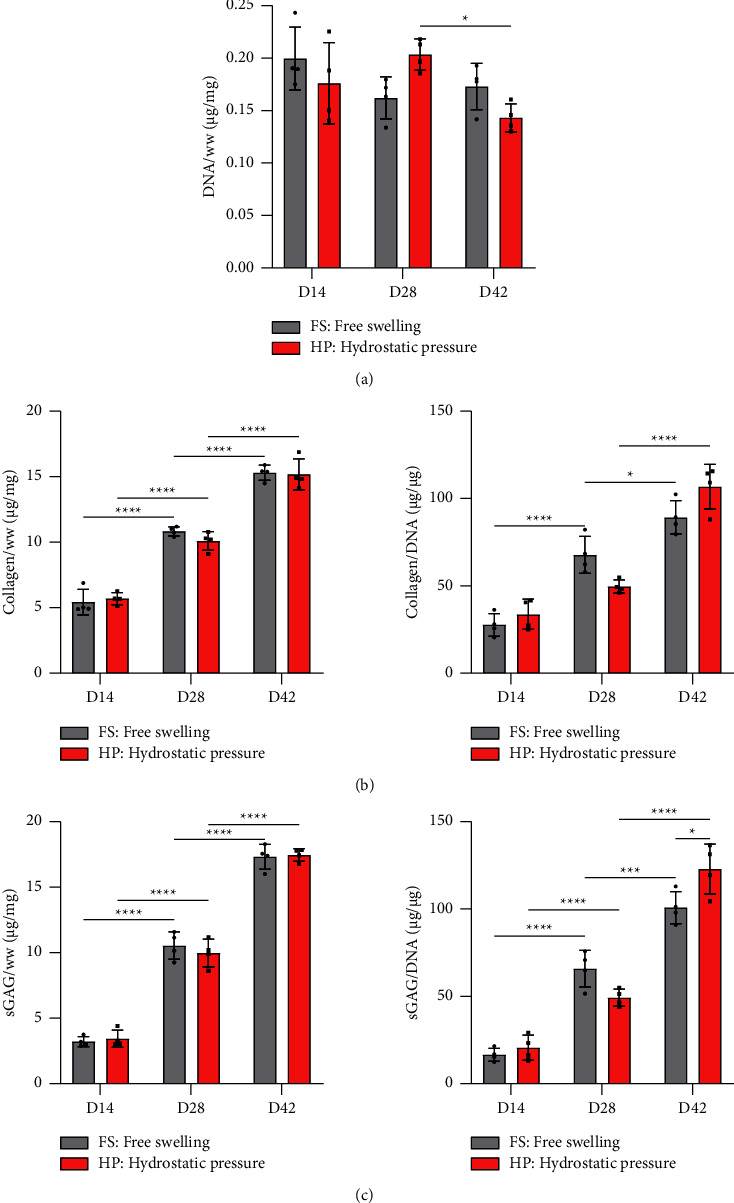
Biochemical content analysis. Influence of the priming period on mechanoresponsiveness of hMSCs. (a) DNA content normalized to wet weight (ww) was determined using Hoechst bisbenzimide H 33258 quantitation assay. (b) Collagen content analysis. Total collagen was determined via hydroxyproline-based quantification assay and normalized to total DNA. (c) sGAG content analysis. sGAG content was determined with DMMB-based assay and normalized to total DNA content. Significant differences are reported using two-way analysis of variance (ANOVA) with a post hoc Tukey's multiple comparison test. All data were represented as mean ± SD; technical replicates *n* ≥ 3, ^*∗*^*p* ≤ 0.05, ^*∗∗*^*p* ≤ 0.01, ^*∗∗∗*^*p* ≤ 0.001, and ^*∗∗∗∗*^*p* ≤ 0.0001.

**Figure 8:**
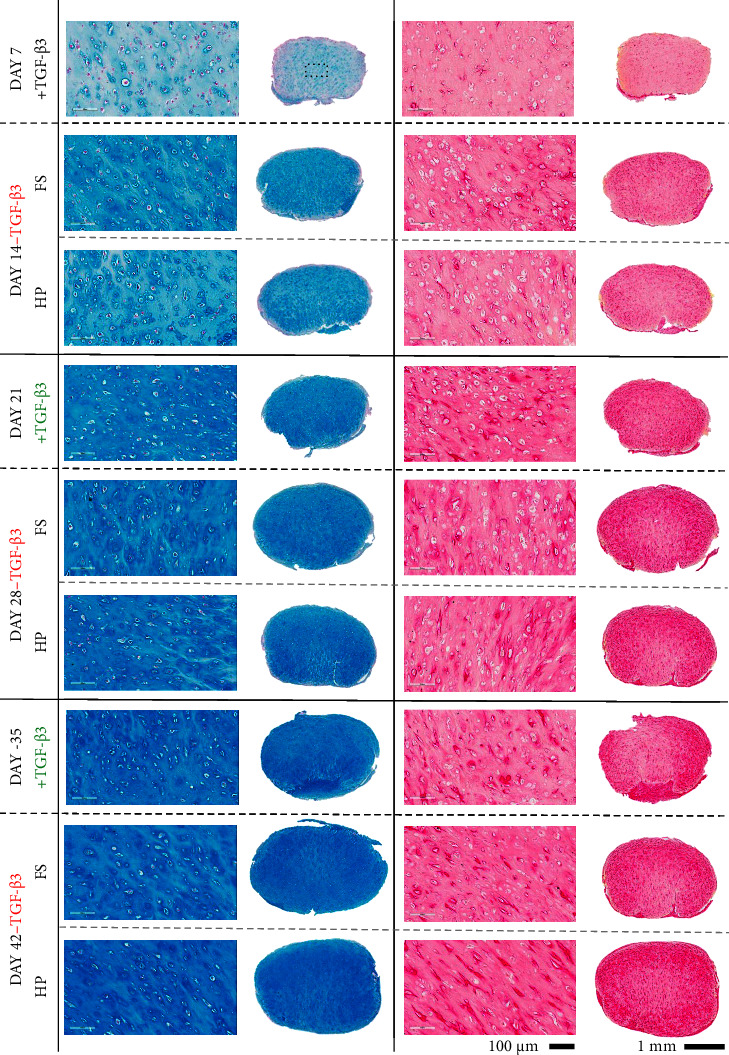
Histological assessment of hMSCs subjected to HP after different priming periods. Alcian blue staining for visualisation of glycosaminoglycans (left panel). Picrosirius red staining for visualisation of collagens (right panel). Dotted rectangle represents the magnified area of the construct.

The application of HP did not appear to affect the majority of examined genes following the removal of TGF-*β*3 from the culture media (Figure 6). *ACAN* expression appeared to be positively affected by HP at days 28 and 42, albeit not statistically significant. Similarly, no major differences in expression of key gene ratios could be observed, except for *ACAN/COL10A1* ratio that was significantly improved at day 42 in the presence of HP. On the protein level, the application of HP after various priming periods did not appear to have major negative or positive effects on synthesis of ECM components (Figure 7). Of note, the application of HP after 35 days of priming led to a significant increase of sGAG/DNA levels at day 42 compared to the free swelling control (Figure 7(c)). The histological assessment of ECM components deposited over the course of 42 days confirms a very robust chondrogenic differentiation, as evidenced by the temporal increase of alcian blue and picrosirius red staining at each examined timepoint (Figure 8). Removal of TGF-*β*3 had no negative effect on the deposition of ECM components at any of the timepoints. Similarly, no discernible differences between mechanically stimulated and corresponding controls could be observed. Generally, alcian blue stain appeared to be more homogeneously distributed than picrosirius red, which stained weaker at the centre of the constructs at later stages of maturation. Alizarin red staining was performed to examine potential calcium deposition (Supplementary Figure 1); no positive staining could be observed.

**Figure 9:**
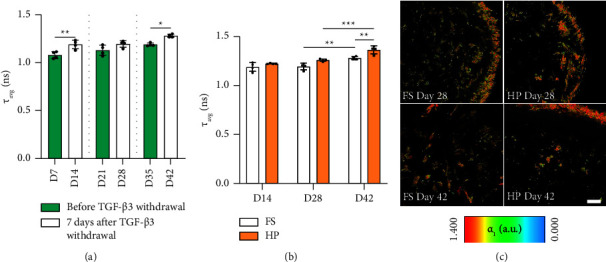
Evaluation of hMSC metabolic activity subjected to HP after priming with TGF-*β*3. The relative metabolic state was determined by using FLIM and quantifying the average lifetime of (*τ*_avg_) of NAD(P)H (see methods). (a) The effect of TGF-*β*3 removal on average lifetime of (*τ*_avg_) of NAD(P)H. (b) The effect of hydrostatic pressure on average lifetime of (*τ*_avg_) of NAD(P)H within chondrogenically primed cartilage grafts. (c) Representative pseudocolour-coded images of free (0.0) and protein-bound (1.4) fraction of NAD(P)H for priming periods of 21 and 35 days. Scale bar = 100 *µ*m. Significant differences are reported using two-way analysis of variance (ANOVA) with a post hoc Tukey's multiple comparison test. All data were represented as mean ± SD; technical replicates *n* ≥ 3, ^*∗*^*p* ≤ 0.05, ^*∗∗*^*p* ≤ 0.01, and ^*∗∗∗*^*p* ≤ 0.001.

Successful differentiation of MSCs is linked to the progressive shift of energy metabolism from glycolysis to oxidative phosphorylation (OxPhos), which can be quantified by determining protein-bound NAD(P)H levels (Figure 9). Continuous static culture in the presence of TGF-*β*3 over 35 days was associated with a gradual increase of average lifetimes of NAD(P)H (*τ*_avg_), indicating a shift towards OxPhos (Figure 2(d)). The removal of the growth factor after 7 and 35 days of priming resulted in a statistically significant increase in *τ*_avg_ values (Figure 9(a)). This effect was further enhanced when HP was applied after 35 days of chondrogenic priming, where *τ*_avg_ values were significantly higher for HP compared to the FS control (Figure 9(b)).

## 4. Discussion

As MSCs undergo chondrogenic differentiation and deposit pericellular and extracellular matrix, their cellular phenotype and associated mechanoresponsiveness might evolve as the function of cell maturity. Several studies have looked at how different priming periods affect chondrogenic differentiation when subsequently subjected to HP; however, employment of different cells, 3D environments, and loading regimes made it challenging to develop a generalized statement regarding suitable priming periods [11]. This study aimed at assessing how chondrogenically primed hMSCs would respond to hydrostatic pressure following specific priming periods in the presence of TGF-*β*3. By culturing hMSCs in the presence of TGF-*β*3 for prespecified periods of time and subsequently removing the growth factor prior to application of mechanical stimulation, we also partially mimicked how such cartilage grafts might behave *in vivo* upon implantation into a mechanically demanding environment. It was found that the removal of TGF-*β*3 differentially impacted the development of engineered grafts in a maturity-dependent manner. Furthermore, only mature engineered cartilage grafts responded positively to the application of HP. Thus, an *in vitro* system for assessing how engineered graft maturity influences their response to mechanical stimulation was established, which provides valuable information to potentially increase the chances of successful outcomes *in viv*o.

Static culture of hMSCs in 2.5% fibrin hydrogels over the course of 35 days of culture resulted in robust chondrogenic differentiation, which was reflected in the continuous upregulation of chondrogenic genes including *COL2A1* and *ACAN*, as well as accumulation of cartilage-specific ECM components. To our surprise, no major increase in the expression of *SOX9* could be observed after day 7 of culture, although the expression of *SOX9* downstream targets such as *ACAN* and *COL2A1* increased continuously until the last examined timepoint at day 35 [26]. This observation could potentially be explained by posttranscriptional as well as posttranslational regulation of *SOX9* and its downstream targets [27]. Gene expression levels do not reveal information regarding the actual protein levels and their activity. It is possible that translation of SOX9 mRNA is inhibited by appropriate miRNAs, and as maturation of MSCs progressed, the silencing was progressively attenuated, thus enhancing translation of SOX9 mRNA [28].

TGF-*β*3 plays a crucial role in various developmental processes including cell differentiation, proliferation, and ECM deposition [29]. Consequently, TGF-*β*3 has established itself as one of the most used growth factors in cartilage tissue engineering to promote chondrogenic differentiation of MSCs [30]. Interestingly, removal of TGF-*β*3 after 7, 21, and 35 days of priming had no major effects on the expression of *SOX9, ACAN*, and *COL2A1,* except for *COL2A1* expression at later timepoints (removal of TGF-*β*3 after 35 days). This capacity of short-term exposure to TGF-*β* to induce robust expression of chondrogenic machinery has previously been demonstrated in specific contexts. For example, it has been shown that transient short-term exposure of cartilage microtissues, generated from hMSCs, to TGF-*β*1 was sufficient to induce robust chondrogenic differentiation [31]. Cumulatively, these observations raise the question whether continuous growth factor supplementation is always necessary for sufficient chondrogenesis and whether fine-tuning of induction kinetics should be leveraged develop novel strategies to achieve hyaline-like phenotype.

Interestingly, the removal of TGF-*β*3 after 35 days of priming results in a decrease of *COL2A1* expression over the next 7 days of culture (compared to day 35), whereas no changes in *ACAN* are registered. There is evidence suggesting that synthesis of collagens in mature chondrocytes is more reliant on the availability of growth factors compared to the synthesis of glycosaminoglycans [32–35]. This might imply that MSCs have acquired a chondrocyte-like state after 35 days of chondrogenic differentiation. Care must be taken when interpreting the implications of this finding, as control constructs where TGF-*β*3 was continuously supplemented into the media over the entire culture period were not included in this study; hence, we cannot be certain whether the same outcome would occur with continuous TGF-*β*3 supplementation. To our surprise, the expression of *COL10A1* appeared to follow the *COL2A1* expression pattern. This contradicts the reported chondroprotective role of this growth factor, which is also reflected in the downregulation of HDAC4 that is responsible for silencing of hypertrophic DNA regions [25, 36]. However, TGF-*β*3 does not signal exclusively through Smad2/3 and has been shown to phosphorylate Smad1/5/8 as well, which is associated with upregulation of hypertrophic and osteogenic markers. Such hypertrophic differentiation represents one of the main challenges of articular cartilage TE using MSCs [37, 38]; therefore, further work is required to identify how specific growth factor priming regimes influences progression along an endochondral pathway. Nevertheless, removal of TGF-*β*3 did not have a negative impact on chondrogenic/hypertrophic gene ratios, suggesting that removal of TGF-*β*3 does not necessarily lead to progression along an endochondral pathway; this was also supported by the relatively weak staining for collagen type X and calcium deposition (Supplementary Figures 1 and 3).

Although the application of HP is generally beneficial during chondrogenic differentiation of MSCs, it is not known whether HP is sufficient to induce differentiation in the absence of exogenous growth factors [11, 39, 40]. Application of HP alone for one week resulted in a statistically significant upregulation of SOX9 compared to the FS control. However, this upregulation was significantly lower (3.5-fold) compared to the growth factor induced increase (40-fold) after one week of culture (Supplementary Figure 2). It is therefore questionable whether HP induced upregulation of SOX9 would be sufficient to initiate expression of its downstream targets.

It is well accepted that application of mechanical cues such as hydrostatic pressure can benefit tissue engineering of hyaline-like cartilage [13, 14]. Interestingly, the extent of the benefit appears to be contextual and dependent on various factors including cell source, culture format, biomaterial, and mode of stimulation [11, 12]. This challenges the identification of the most appropriate *in vitro* bioreactor conditions to enhance differentiation and ECM deposition. For example, previous studies from our lab have shown that porcine MSCs embedded within agarose and fibrin hydrogels respond positively to the application of HP [14, 41]. However, when human MSCs were encapsulated into fibrin hydrogels, no benefits of HP application could be identified [16, 42]. One possible explanation for such a discrepancy could be the employment of different HP magnitudes (10 vs 2 MPa). However, lower magnitudes have been previously shown to support chondrogenesis in a collagen scaffold and in pellet cultures employing human MSCs [39, 42–44]. This points to intricate interdependencies that need to be identified and leveraged to enable the engineering of hyaline-like cartilage using stem/progenitor cell populations.

In this context, there is also very little knowledge regarding the impact of HP on hMSC fate after specific chondrogenic priming periods [14, 15, 45, 46]. Based on the quantitative gene expression data, neither negative nor positive effects of HP application could be observed for most of the examined genes at any timepoints. *ACAN* was the only gene that appeared to respond positively to mechanical stimulation after 35 days of priming. This observation was further confirmed by the biochemical content analysis that revealed significant increase of sGAG/DNA at these later timepoints. An evaluation of chondrogenic/hypertrophic gene ratios also indicates that mechanical stimulation is supportive of the chondrogenic phenotype after longer priming periods. Cumulatively, this data suggests that priming/culture periods and the “maturity” of chondrogenically primed hMSCs can play a considerable role in how mechanical cues are perceived by differentiating cells. These results are in line with observations made *in vivo*, where delayed application of stress was most beneficial in terms of regeneration [7, 8, 10].

It has been reported that maturation of MSCs is linked to metabolic changes, where the glycolytic mode of energy generation shifts towards oxidative phosphorylation (OxPhos) [47]. We could confirm the incremental increase of protein-bound NAD(P)H levels over the course of 35 days in static culture using FLIM [48]. Although mature chondrocytes are known to utilize glycolysis in their hypoxic niche for energy generation, this glycolytic state appears to be more pronounced in naïve MSCs [49]. Interestingly, the removal of TGF-*β*3 appeared to facilitate the shift towards OxPhos at all examined timepoints. This indicates that chondrogenesis-associated metabolic shifts can be induced by short-term exposure to growth factors and should be considered during the optimization of chondrogenic culture protocols. More importantly, the application of HP at later timepoints appeared to further progress the metabolic shift towards OxPhos, which has been associated with maturation of MSCs and has been shown to reduce downstream functional features of osteoarthritis [47, 50]. In summary, this points to the essential role of mechanical cues in the maintenance of the chondrogenic phenotype in differentiating MSCs in a maturity-dependent manner.

## 5. Conclusion

Herein we have established an experimental platform that partially mimics the transition of a cartilage graft from an *in vitro* culture into a mechanically stimulated *in vivo* environment. This was achieved by culturing fibrin embedded hMSCs in chondrogenic media with TGF-*β*3 and subsequently subjecting these constructs to hydrostatic pressure following the removal of exogenous growth factors. By utilizing 1-, 3-, and 5-week priming periods, we have examined how the maturation state of chondrogenically primed MSCs affects their response to HP. Subjecting cells to HP was only beneficial at later stages of differentiation, which was reflected by an upregulation of ACAN, sGAG deposition, and metabolic activity upon mechanical stimulation. These results suggest that in this specific experimental setup a priming period of at least 5 weeks would be advisable before *in vivo* implantation of such an engineered cartilage graft.

## Figures and Tables

**Table 1:** List of employed primers.

Gene name	Forward/reverse
*SOX9 (SRY-box transcription factor 9)*	F: 5′-CTCTGGAGACTTCTGAACG R: 5′-AGATGTGCGTCTGCTC

*RUNX2 (runt-related transcription factor 2)*	F: 5′-AAGCTTGATGACTCTAAACC R: 5′-TCTGTAATCTGACTCTGTCC

*COL2A1 (collagen type II alpha 1 chain)*	F: 5′-GAAGAGTGGAGACTACTG R: 5′-CAGATGTGTTTCTTCTCCTG

*ACAN (aggrecan)*	F: 5′-CACCCCATGCAATTTGAG R: 5′-AGATCATCACCACACAGTC

*COL10A1 (collagen type X alpha 1 chain)*	F: 5′-GCTAGTATCCTTGAACTTGGR: 5′-CCTTTACTCTTTATGGTGTAGG

*B2M (beta-2-microglobulin)*	F: 5′-AAGGACTGGTCTTTCTATCTC R: 5′-GATCCCACTTAACTATCTTGG

*HDAC4 (histone deacetylase 4)*	F: 5′-CAAGAACAAGGAGAAGGGCAAAG R: 5′-GGACTCTGGTCAAGGGAACTG

*COL6A1 (collagen type VI alpha 1 chain)*	F: 5′-GACCTCGGACCTGTTGGGTAC R: 5′-TACCCCATCTCCCCCTTCAC

## Data Availability

The data used in this study are available on request from the author.

## Supplementary Materials

Supplementary Materials Supplementary Figure 1: Alizarin red (AR) 1% w/v at pH 4.1 was used to assess calcium deposition (all Sigma-Aldrich). All stained slides were imaged with Aperio ScanScope (Leica Biosystems). Supplementary Figure 2: 10 ng cDNA of each sample was used for real-time PCR. Amplification was performed using SYBRTM Select Master Mix and 7500 Fast System (Applied Biosystems™). B2M functioned as a housekeeping gene. KiCqStart® SYBR® Green predesigned primers were purchased from Sigma. Supplementary Figure 3: Primary antibodies Col10a1 (AB49945, 1 : 200) were incubated overnight at 4°C. Goat anti-mouse IgM (AB150121) was used for Col10a1 sections (4 hours at RT); nuclei were counterstained with DAPI (2 *μ*g/mL, Sigma-Aldrich). Presence of collagen type X was visualized using Leica SP8 scanning confocal microscope.

## References

[1] Mankin H. J., Dorfman H., Lippiello L., Zarins A. (1971). Biochemical and metabolic abnormalities in articular cartilage from osteo-arthritic human hips: II. Correlation of morphology with biochemical and metabolic data. *The Journal of Bone and Joint Surgery*.

[2] Armiento A. R., Stoddart M. J., Alini M., Eglin D. (2018). Biomaterials for articular cartilage tissue engineering: learning from biology. *Acta Biomaterialia*.

[3] Makris E. A., Gomoll A. H., Malizos K. N., Hu J. C., Athanasiou K. A. (2015). Repair and tissue engineering techniques for articular cartilage. *Nature Reviews Rheumatology*.

[4] Johnstone B., Hering T. M., Caplan A. I., Goldberg V. M., Yoo J. U. (1998). In vitro chondrogenesis of bone marrow-derived mesenchymal progenitor cells. *Experimental Cell Research*.

[5] Arvind V., Huang A. H. (2017). Mechanobiology of limb musculoskeletal development. *Annals of the New York Academy of Sciences*.

[6] Einhorn T. A., Gerstenfeld L. C. (2015). Fracture healing: mechanisms and interventions. *Nature Reviews Rheumatology*.

[7] Boerckel J. D., Kolambkar Y. M., Stevens H. Y., Lin A. S. P., Dupont K. M., Guldberg R. E. (2012). Effects of in vivo mechanical loading on large bone defect regeneration. *Journal of Orthopaedic Research*.

[8] Gardner M. J., van der Meulen M. C. H., Demetrakopoulos D. (2006). In vivo cyclic axial compression affects bone healing in the mouse tibia. *Journal of Orthopaedic Research*.

[9] Steck E., Fischer J., Lorenz H., Gotterbarm T., Jung M., Richter W. (2009). Mesenchymal stem cell differentiation in an experimental cartilage defect: restriction of hypertrophy to bone-close neocartilage. *Stem Cells and Development*.

[10] He A., Liu L., Luo X. (2017). Repair of osteochondral defects with in vitro engineered cartilage based on autologous bone marrow stromal cells in a swine model. *Scientific Reports*.

[11] Pattappa G., Zellner J., Johnstone B., Docheva D., Angele P. (2019). Cells under pressure- the relationship between hydrostatic pressure and mesenchymal stem cell chondrogenesis. *European Cells and Materials*.

[12] Aprile P., Kelly D. J. (2020). Hydrostatic pressure regulates the volume, aggregation and chondrogenic differentiation of bone marrow derived stromal cells. *Frontiers in Bioengineering and Biotechnology*.

[13] Steward A. J., Thorpe S. D., Vinardell T., Buckley C. T., Wagner D. R., Kelly D. J. (2012). Cell‐matrix interactions regulate mesenchymal stem cell response to hydrostatic pressure. *Acta Biomaterialia*.

[14] Carroll S. F., Buckley C. T., Kelly D. J. (2014). Cyclic hydrostatic pressure promotes a stable cartilage phenotype and enhances the functional development of cartilaginous grafts engineered using multipotent stromal cells isolated from bone marrow and infrapatellar fat pad. *Journal of Biomechanics*.

[15] Meyer E. G., Buckley C. T., Steward A. J., Kelly D. J. (2011). The effect of cyclic hydrostatic pressure on the functional development of cartilaginous tissues engineered using bone marrow derived mesenchymal stem cells. *Journal of the Mechanical Behavior of Biomedical Materials*.

[16] Chariyev-Prinz F., Szojka A., Neto N., Burdis R., Monaghan M. G., Kelly D. J. (2023). An assessment of the response of human MSCs to hydrostatic pressure in environments supportive of differential chondrogenesis. *Journal of Biomechanics*.

[17] Arlyng G. G., Lia A. B., Bennett K. E., Casey S. M., Pieter A. J. B., Fergal J. (2021). Systematic comparison of biomaterials-based strategies for osteochondral and chondral repair in large animal models. *Advanced Healthcare Materials*.

[18] Anderson D. E., Markway B. D., Bond D., McCarthy H. E., Johnstone B. (2016). Responses to altered oxygen tension are distinct between human stem cells of high and low chondrogenic capacity. *Osteoarthritis and Cartilage*.

[19] Daly A. C., Sathy B. N., Kelly D. J. (2018). Engineering large cartilage tissues using dynamic bioreactor culture at defined oxygen conditions. *Journal of Tissue Engineering*.

[20] Lafont J. E. (2010). Lack of oxygen in articular cartilage: consequences for chondrocyte biology. *International Journal of Experimental Pathology*.

[21] Pattappa G., Johnstone B., Zellner J., Docheva D., Angele P. (2019). The importance of physioxia in mesenchymal stem cell chondrogenesis and the mechanisms controlling its response. *International Journal of Molecular Sciences*.

[22] Kafienah W., Sims T. J., Swansbury J. (2003). Biochemical methods for the analysis of tissue-engineered cartilage. *Cancer Cytogenetics. Methods and Protocols*.

[23] Neto N. G. B., O’Rourke S. A., Zhang M., Fitzgerald H. K., Dunne A., Monaghan M. G. (2022). Non-invasive classification of macrophage polarisation by 2P-FLIM and machine learning. *Elife*.

[24] Perottoni S., Neto N. G. B., Di Nitto C., Dmitriev R. I., Raimondi M. T., Monaghan M. G. (2021). Intracellular label-free detection of mesenchymal stem cell metabolism within a perivascular niche-on-a-chip. *Lab on a Chip*.

[25] Winbanks C. E., Wang B., Beyer C. (2011). TGF-Β regulates miR-206 and miR-29 to control myogenic differentiation through regulation of HDAC4. *Journal of Biological Chemistry*.

[26] Bi W., Deng J. M., Zhang Z., Behringer R. R., de Crombrugghe B. (1999). Sox9 is required for cartilage formation. *Nature Genetics*.

[27] Allas L., Boumédiene K., Baugé C. (2019). Epigenetic dynamic during endochondral ossification and articular cartilage development. *Bone*.

[28] Iaquinta M. R., Lanzillotti C., Mazziotta C. (2021). The role of microRNAs in the osteogenic and chondrogenic differentiation of mesenchymal stem cells and bone pathologies. *Theranostics*.

[29] Hill C. S. (2016). Transcriptional control by the SMADs. *Cold Spring Harbor Perspectives in Biology*.

[30] Grafe I., Alexander S., Peterson J. R. (2018). TGF-Β family signaling in mesenchymal differentiation. *Cold Spring Harbor Perspectives in Biology*.

[31] Futrega K., Robey P. G., Klein T. J., Crawford R. W., Doran M. R. (2021). A single day of TGF-*β*1 exposure activates chondrogenic and hypertrophic differentiation pathways in bone marrow-derived stromal cells. *Communications Biology*.

[32] Bachmann B., Spitz S., Schädl B. (2020). Stiffness matters: fine-tuned hydrogel elasticity alters chondrogenic redifferentiation. *Frontiers in Bioengineering and Biotechnology*.

[33] Darling E. M., Athanasiou K. A. (2005). Growth factor impact on articular cartilage subpopulations. *Cell and Tissue Research*.

[34] MacBarb R. F., Makris E. A., Hu J. C., Athanasiou K. A. (2013). A chondroitinase-ABC and TGF-*β*1 treatment regimen for enhancing the mechanical properties of tissue engineered fibrocartilage. *Acta Biomaterialia*.

[35] Mauck R. L., Yuan X., Tuan R. S. (2006). Chondrogenic differentiation and functional maturation of bovine mesenchymal stem cells in long-term agarose culture. *Osteoarthritis and Cartilage*.

[36] Spagnoli A., O’Rear L., Chandler R. L. (2007). TGF-beta signaling is essential for joint morphogenesis. *The Journal of Cell Biology*.

[37] Finnson K. W., Parker W. L., ten Dijke P., Thorikay M., Philip A. (2008). ALK1 opposes ALK5/smad3 signaling and expression of extracellular matrix components in human chondrocytes. *Journal of Bone and Mineral Research*.

[38] Wrighton K. H., Lin X., Yu P. B., Feng X.-H. (2009). Transforming growth factor *β* can stimulate Smad1 phosphorylation independently of bone morphogenic protein receptors. *Journal of Biological Chemistry*.

[39] Miyanishi K., Trindade M. C. D., Lindsey D. P. (2006). Dose- and time-dependent effects of cyclic hydrostatic pressure on transforming growth factor-*β*3-induced chondrogenesis by adult human mesenchymal stem cells in vitro. *Tissue Engineering*.

[40] Puetzer J., Williams J., Gillies A., Bernacki S., Loboa E. G. (2013). The effects of cyclic hydrostatic pressure on chondrogenesis and viability of human adipose- and bone marrow-derived mesenchymal stem cells in three-dimensional agarose constructs. *Tissue Engineering Part A*.

[41] Steward A. J., Wagner D., Kelly D. J. (2013). The pericellular environment regulates cytoskeletal development and the differentiation of mesenchymal stem cells and determines their response to hydrostatic pressure. *European Cells and Materials*.

[42] Angele P., Yoo J. U., Smith C. (2003). Cyclic hydrostatic pressure enhances the chondrogenic phenotype of human mesenchymal progenitor cells differentiated in vitro. *Journal of Orthopaedic Research*.

[43] Ogawa R., Mizuno S., Murphy G. F., Orgill D. P. (2009). The effect of hydrostatic pressure on three-dimensional chondroinduction of human adipose-derived stem cells. *Tissue Engineering Part A*.

[44] Lindsey D. P., Li K. W., Tummala P. (2008). Hydrostatic pressure enhances chondrogenic differentiation of human bone marrow stromal cells in osteochondrogenic medium. *Annals of Biomedical Engineering*.

[45] Li Y., Zhou J., Yang X., Jiang Y., Gui J. (2016). Intermittent hydrostatic pressure maintains and enhances the chondrogenic differentiation of cartilage progenitor cells cultivated in alginate beads. *Development Growth and Differentiation*.

[46] Liu Y., Buckley C. T., Downey R., Mulhall K. J., Kelly D. J. (2012). The role of environmental factors in regulating the development of cartilaginous grafts engineered using osteoarthritic human infrapatellar fat pad–derived stem cells. *Tissue Engineering Part A*.

[47] Lee A. R., Moon D. K., Siregar A. (2019). Involvement of mitochondrial biogenesis during the differentiation of human periosteum-derived mesenchymal stem cells into adipocytes, chondrocytes and osteocytes. *Archives of Pharmacal Research*.

[48] Blacker T. S., Mann Z. F., Gale J. E. (2014). Separating NADH and NADPH fluorescence in live cells and tissues using FLIM. *Nature Communications*.

[49] Lane R. S., Fu Y., Matsuzaki S., Kinter M., Humphries K. M., Griffin T. M. (2015). Mitochondrial respiration and redox coupling in articular chondrocytes. *Arthritis Research and Therapy*.

[50] Ohashi Y., Takahashi N., Terabe K. (2021). Metabolic reprogramming in chondrocytes to promote mitochondrial respiration reduces downstream features of osteoarthritis. *Scientific Reports*.

