# Supplementary material for: Time-Dependent Anabolic Response of hMSC-Derived Cartilage Grafts to Hydrostatic Pressure
Source: J Tissue Eng Regen Med. 2023 Aug 23;2023:9976121. doi: 10.1155/2023/9976121 (PMC11919143; doi:10.1155/2023/9976121)
Supplement: Supplementary Materials — Supplementary Figure 1: Alizarin red (AR) 1% w/v at pH 4.1 was used to assess calcium deposition (all Sigma-Aldrich). All stained slides were imaged with Aperio ScanScope (Leica Biosystems). Supplementary Figure 2: 10 ng cDNA of each sample was used for real-time PCR. Amplification was performed using SYBRTM Select Master Mix and 7500 Fast System (Applied Biosystems™). B2M functioned as a housekeeping gene. KiCqStart® SYBR® Green predesigned primers were purchased from Sigma. Supplementary Figure 3: Primary antibodies Col10a1 (AB49945, 1 : 200) were incubated overnight at 4°C. Goat anti-mouse IgM (AB150121) was used for Col10a1 sections (4 hours at RT); nuclei were counterstained with DAPI (2 μg/mL, Sigma-Aldrich). Presence of collagen type X was visualized using Leica SP8 scanning confocal microscope. [file 9976121.f1.pdf]

Supplementary information

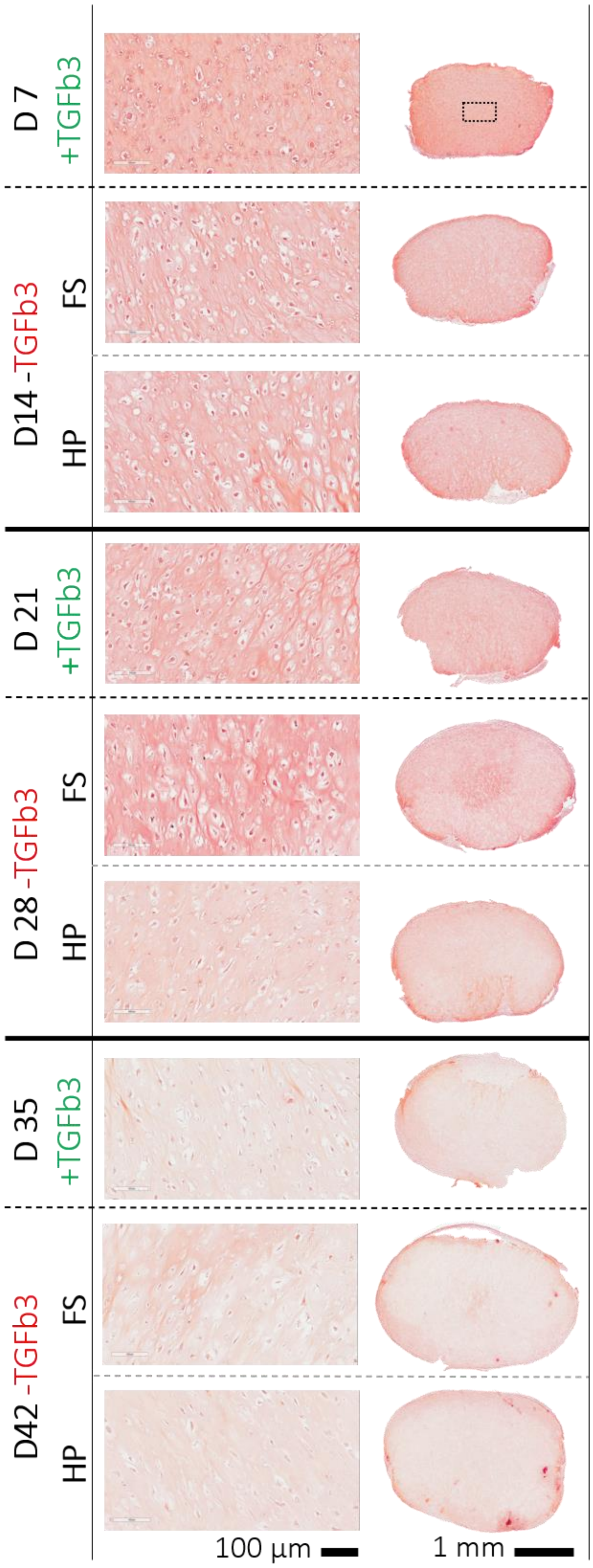

Supplementary Figure 1: **Histological analysis of calcium deposition.** Alizarin red (AR) staining was used to assess progression towards endochondral ossification. Dotted rectangle represents the magnified area of the construct.

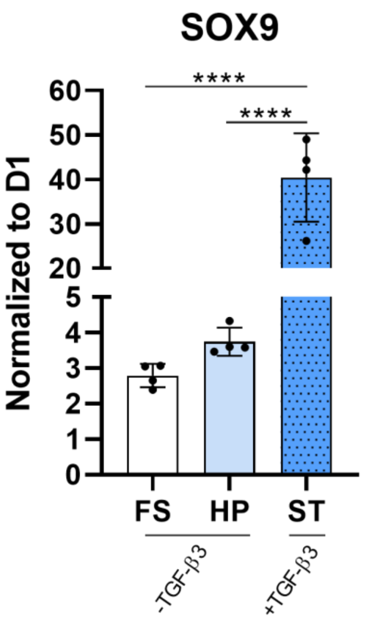

Supplementary Figure 2: HP induction of chondrogenesis in absence of growth factors. A) Relative gene expression of SOX9 was normalised to expression levels at the start of the 7-day experiment (Day 1). Relative expression levels were determined using $2^{-\Delta\Delta Ct}$ method. Housekeeping gene: B2M. Significant differences are reported using unpaired two-tailed Student's t-test. All data represented as mean ± SD; technical replicates n=3; *p≤0.05; **p≤0.01; ***p≤0.001.

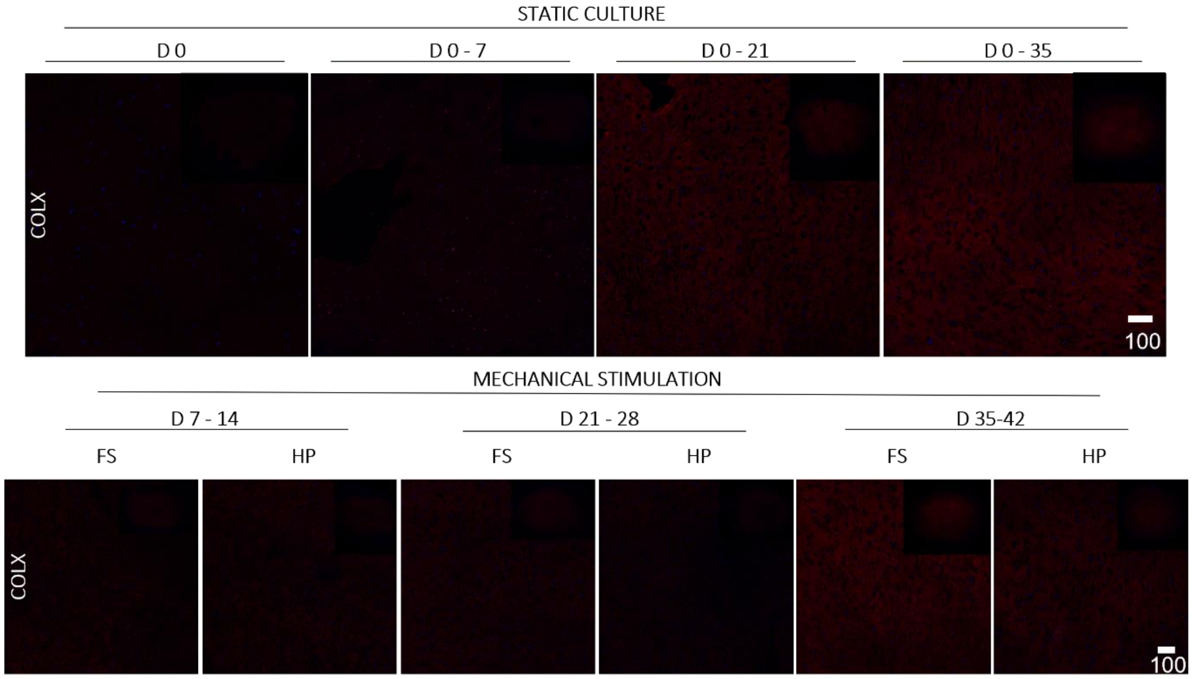

Supplementary Figure 3: Immunofluorescence analysis of collagen types X during static culture and subsequent HP application at different timepoints. Confocal microscopy images for collagen type X (red). Free swelling (FS) was used as control. Nuclei counterstained blue (DAPI). Scale bar = 100 µm.
